# Nonlinear Radiation Heat Transfer Effects in the Natural Convective Boundary Layer Flow of Nanofluid Past a Vertical Plate: A Numerical Study

**DOI:** 10.1371/journal.pone.0103946

**Published:** 2014-09-24

**Authors:** Meraj Mustafa, Ammar Mushtaq, Tasawar Hayat, Bashir Ahmad

**Affiliations:** 1 School of Natural Sciences (SNS), National University of Sciences and Technology (NUST), Islamabad, Pakistan; 2 Research Centre for Modeling and Simulation (RCMS), National University of Sciences and Technology (NUST), Islamabad, Pakistan; 3 Department of Mathematics, Quaid-I-Azam University, Islamabad, Pakistan; 4 Department of Mathematics, Faculty of Science, King Abdulaziz University, Jeddah, Saudi Arabia; University of Zurich, Switzerland

## Abstract

The problem of natural convective boundary layer flow of nanofluid past a vertical plate is discussed in the presence of nonlinear radiative heat flux. The effects of magnetic field, Joule heating and viscous dissipation are also taken into consideration. The governing partial differential equations are transformed into a system of coupled nonlinear ordinary differential equations via similarity transformations and then solved numerically using the Runge–Kutta fourth-fifth order method with shooting technique. The results reveal an existence of point of inflection for the temperature distribution for sufficiently large wall to ambient temperature ratio. Temperature and thermal boundary layer thickness increase as Brownian motion and thermophoretic effects intensify. Moreover temperature increases and heat transfer from the plate decreases with an increase in the radiation parameter.

## Introduction

Solar energy is probably the most suitable source of renewable energy that can meet the current energy requirements. The energy obtained from nature in the form of solar radiations can be directly transformed into heat and electricity. The idea of using small particles to collect solar energy was first investigated by Hunt [1] in the 1970s. Researchers concluded that with the addition of nanoparticles in the base fluids, heat transfer and the solar collection processes can be improved. Masuda et al. [2] discussed the alteration of thermal conductivity and viscosity by dispersing ultra-fine particles in the liquid. Choi and Eastman [3] were the first to introducethe terminology of nanofluids when they experimentally discovered an effective way of controlling heat transfer rate using nanoparticles. Buongiorno [4] developed the nonhomogeneous equilibrium mathematical model for convective transport of nanofluids. He concluded that Brownian motion and thermophoretic diffusion of nanoparticles are the most important mechanisms for the abnormal convective heat transfer enhancement. The relevant processes are briefly described in [5]–[7]. Investigations in the nanofluid flows have received remarkable popularity in research community in last couple of decades primarily due to their variety of applications in power generation, in transportation where nanofluid may be utilized in vehicles as coolant, shock absorber, fuel additives etc., in cooling and heating problems which may involve the use of nanofluids for cooling of microchips in computer processors, in improving performance efficiency of refrigerant/air-conditioners etc. and in biomedical applications in which magnetic nanoparticles may be used in medicine, cancer therapy and tumor analysis. Recently the researchers have proposed the idea of using solar collector based nanofluids for optimal utilization of solar energy radiation [8], [9]. Buongiorno and Hu [10] discussed the heat transfer enhancement via nanoparticles for nuclear reactor application. Huminic and Huminic [11] showed that use of nanofluids in heat exchangers has advantage in the energy efficiencyand it leads to better system performance.

Nield and Kuznetsov [12] and Kuznetsov and Nield [13] studied Cheng-Minkowycz problem of natural convective boundary layer flow in a porous medium saturated by a nanofluid and natural convective boundary-layer flow of a nanofluid past a vertical plate respectively. Khan and Pop [14] obtained the numerical solution for two-dimensional flow of nanofluid over a linearly stretching sheet using Keller-box method. Makinde and Aziz [15] extended the work of Khan and Pop [14] by implementing convective boundary condition for solving energy equation. Mustafa et al. [16] obtained series solution for stagnation-point flow of a nanofluid by using homotopy analysis method (HAM). Ahmad et al. [17] considered the classical Blasius and Sakiadis problems in nanofluids. Rana and Bhargava [18] examined the flow of nanofluid past a nonlinearly stretching sheet by finite element method. Numerical solution for nanofluid flow past a stretching cylinder with non- uniform heat source/sink was considered by Rasekh et al. [19]. Mustafa et al. [20] used HAM to explore the two-dimensional flow of nanofluid due to a convectively heated exponential stretching surface. In another paper, Mustafa et al. [21] provided both numerical and analytic solutions for exponentially stretched flow of nanofluid. Mustafa et al. [22] also considered an unsteady boundary layer flow of nanofluid past an impulsively stretching sheet and obtained the analytic solution using HAM. Rashidi et al. [23] discussed the steady flow of viscous nanofluid with entropy generation. Here the flow is generated due to rotating porous disk. Ashorynejad et al. [24] investigated nanofluid flow over stretching cylinder in the presence of magnetic field. The effect of magnetic force on the boundary layer flow of nanofluid past a linearly stretching sheet has been reported by Ibrahim et al. [25]. Analytical solution of magneto-nanofluid flow was reported by Sheikholeslami et al. [26]. Sheikholeslami et al. [27] also investigated the nanofluid flow with heat transfer in the presence of an applied magnetic field.

It has been seen that the buoyancy forces stemming from the heating or cooling of the continuously moving surface alter the flow and thermal fields and thereby the heat transfer characteristics of the manufacturing process [28], [29]. The process of natural convection has been thoroughly explained in [30]. Aziz and Khan [31] solved the problem of natural convective flow of a nanofluid past a vertical plate with convective boundary conditions. Uddin et al. [32] obtained numerical solution for steady two dimensional MHD free convective boundary layer flow of an electrically conducting nanofluid past a vertical flat plate with Newtonian heating boundary condition. Kuznetsov and Nield [33], [34] revised their classical natural convection flow problems in nanofluids [12], [13] by taking into account zero nanoparticle wall mass flux condition. Recently MHD mixed convection stagnation-point flow of nanofluid past a convectively heated stretching/shrinking sheet was analyzed by Makinde et al. [35]. Mustafa et al. [36] investigated the effect of an induced magnetic field on the mixed convection peristaltic motion of nanofluid in a vertical channel. Turkyilmazoglu and Pop [37] examined the heat and mass transfer effects in an unsteady convection flow of nanofluid in the presence of thermal radiation. Unsteady convection flow of nanofluids past a vertical plate was studied by Turkyilmazoglu [38].

Raptis and Perdikis [39] studied the viscoelastic fluid motion under the influence of radiations. Seddeek [40] investigated the free convection problem past a semi-infinite plate with magnetic field, radiation and variable viscosity effects. Cortell [41] numerically solved the Sakiadis flow problem by considering thermal radiations. Influence of thermal radiation on the Blasius flow of second grade fluid has been addressed by Hayat et al. [42]. Magyari and Pantokratoras [43] showed that the linear radiation problem can be simply reduced to re-scaling of Prandtl number by a factor containing the radiation parameter. Unsteady flow of nanofluid under the influence of MHD and thermal radiation has been reported by Khan et al. [44]. Motsumi and Makinde [45] investigated the effects of radiation and viscous dissipation on the boundary layer flow of a nanofluid over a permeable flat plate. Radiation effect on nanofluid flow above nonlinearly stretching sheet has been considered by Hady et al. [46]. Marangoni convection boundary layer flow has been investigated by Mat et al. [47]. Effect of incident radiation due to solar energy in an incompressible flow of nanofluid has been presented by Kandasamy et al. [48]. Heat transfer enhancement in the unsteady Hiemenz flow of nanofluid past a wedge has been discussed by Mohamad et al. [49]. The above mentioned studies were confined to the linear radiative heat transfer effects which are only valid for small temperature differences. The idea of nonlinear radiation heat transfer has recently been presented by some researchers (see Pantokratoras and Fang [50], Mushtaq et al. [51], Cortell [52] and Mushtaq et al. [53].

The present work deals with the natural convection flow of an electrically conducting nanofluid past a vertical plate in the presence of nonlinear thermal radiation. Effects of Joule heating and viscous dissipation are also taken into account. Mathematical model proposed by Buongiorno [4] is adopted. The dimensionless mathematical problems have been dealt by shooting method with fourth-fifth-order-Runge-Kutta integration scheme. In shooting method, the boundary conditions are considered as a multivariate function of the initial conditions at some point. It takes advantage of the faster convergence and simple implementation of the methods for initial value problems such as fourth-fifth-order Runge-Kutta (RK45) method, Euler method etc. Due to this reason it has been frequently applied now days to give numerical solutions of various complicated nonlinear boundary value problems (see for instance [15], [20], [25], [32], [41], [51], [53] etc.). Graphs are presented to see the physical behaviors of various interesting parameters. In addition the numerical results of wall heat and mass transfer rates are discussed in detail.

## Problem Formulation

**Figure 1:**
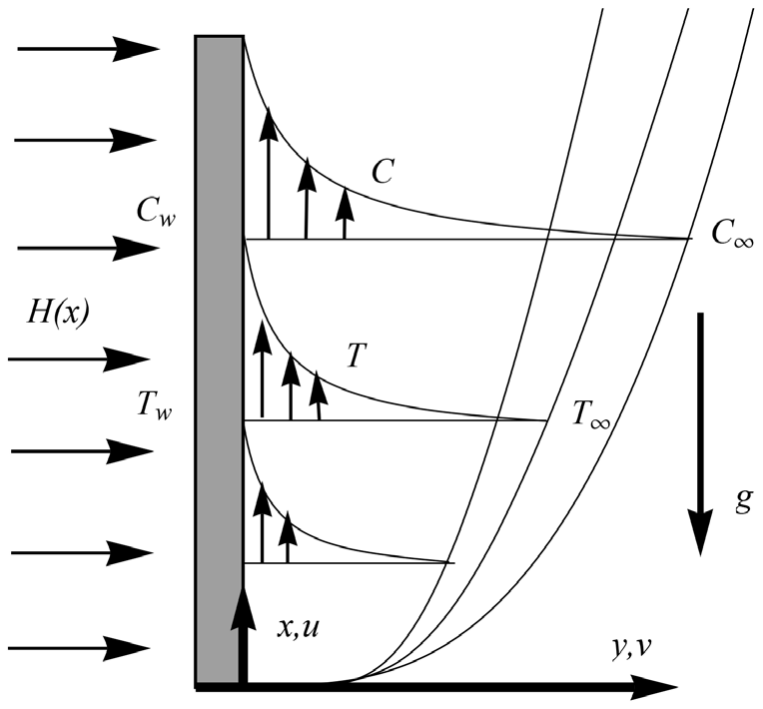
Sketch of the problem and coordinate system. Here 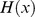 is the variable magnetic field, 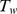 is the constant wall temperature, 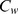 is the nanoparticle wall concentration, 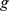 is the gravitational acceleration and 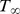 and 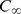 are the ambient temperature and nanoparticle concentration respectively.



We consider laminar two-dimensional boundary layer flow of nanofluid past a vertical plate located at 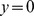. The magnetic field of strength 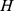 is applied in the 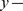 direction whereas the induced magnetic field is neglected subject to the assumption of small magnetic Reynolds number. Heat transfer analysis is carried out in the presence of nonlinear thermal radiation, Joule heating and viscous dissipation effects. The plate is maintained at constant temperature 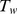 whereas 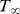 denotes the ambient fluid’s temperature (see Fig. 1). Using the standard boundary layer assumptions and the Oberbeck-Boussinesq approximation we obtain the following boundary layer equations governing the steady incompressible flow of nanofluid (see Nield and Kuznetsov [12], Kuznetsov and Nield [13] and Mushtaq et al. [53]).

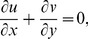
(1)




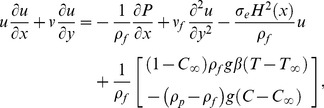
(2)




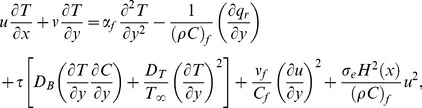
(3)




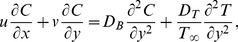
(4)where 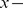 and 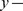 are the coordinates along and normal to the plate respectively, 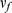 is the kinematic viscosity, 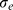 is the electrical conductivity of fluid, 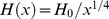 is variable magnetic field, 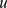 and 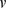 are the velocity components along the 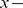 and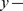 directions respectively, 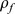 and 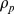 are the densities of base fluid and nanoparticle material respectively, *g* is acceleration due to gravity, 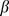 is volumetric thermal expansion coefficient of base fluid, 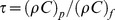 is the ratio of effective heat capacity of the nanoparticle material to the effective heat capacity of the base fluid, 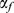 is thermal diffusivity of base fluid, 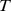 is the local temperature, 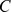 is local volume fraction of nanoparticles, 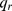 is nonlinear radiative heat flux, 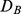 is Brownian diffusion coefficient and 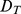 is thermophoretic diffusion coefficient. The boundary conditions in the present problem are:
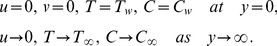
(5)


The radiative heat flux expression in Eq. (3) is given by the Rosseland approximation [54] as 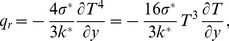
(6)where 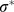 and 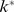 are the Stefan-Boltzman constant and the mean absorption coefficient respectively. Eq. (6) results in a highly nonlinear energy equation in 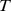 and it is difficult to obtain its solution. However, researchers have solved this problem in past by assuming small temperature differences within the flow (see [39]–[49]). In this situation, Rosseland formula can be linearized about ambient temperature 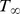. This means to simply replace 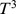 in Eq. (6) with 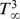. Now (3) can be expressed as:
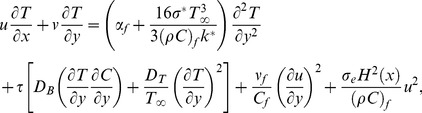
(7)


However if we avoid above mentioned assumption the radiative heat flux in Eq. (3) results in a highly nonlinear radiation expression which is the subject of current study. Hence the energy equation for nonlinear thermal radiation will be:
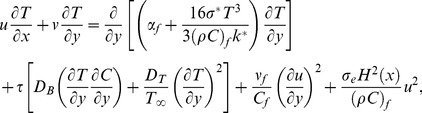
(8)


Using the dimensionless variables [31]

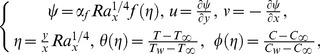
(9)with 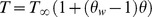 and 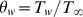 (temperature ratio parameter with 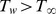), Eq. (1) is identically satisfied and the Eqs. (2), (4), (7) and (8) yield the following differential equations



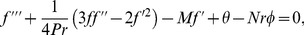
(10)

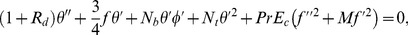
(11)

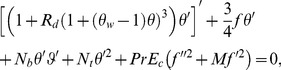
(12)

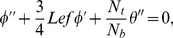
(13)where 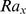 is the local Rayleigh number, 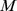 is the magnetic parameter, 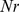 is the buoyancy ratio parameter, 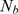 is Brownian motion parameter, 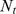 is the thermophoresis parameter, 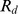 is the radiation parameter, 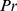 is the Prandtl number, 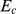 is the Eckert number and 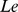 is the Lewis number. These parameters are defined as under:
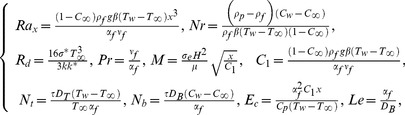
(14)and the transformed boundary conditions are
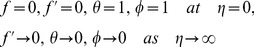
(15)


The quantities of practical interest in this study are the local skin friction coefficient 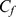, the local Nusselt number 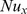 and the local Sherwood number 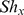 which are defined as:
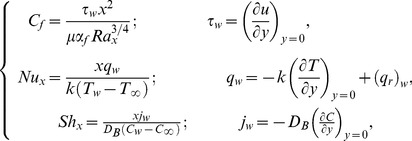
(16)where 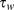 is the wall shear stress and 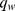 and 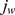 are wall heat and mass fluxes respectively. Following Kuznetsov and Nield [13] the simplified form of above expressions is as under:
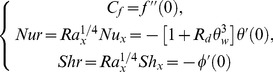
(17)


## Numerical Results and Discussion

The dimensionless mathematical problems given in Eqs. (10)–(13) with the boundary conditions (15) have been solved for the numerical solutions via shooting method. The code for shooting method with fourth-fifth-order Runge-Kutta integration using adaptive step size has been developed in MATLAB. The computations have been performed on 64 bit Core i-7 machine with 8GB RAM. To assess the accuracy of code, the results are compared with Kuznetsov and Nield [13] in a limiting case and found in very good agreement (see Table 1). Table 2 provides the numerical values of wall temperature gradient and wall concentration gradient for different values of 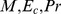 and 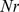 with the other parameters fixed. It may be noted here that values of parameters 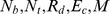 and 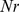 characterize the strengths of Brownian motion, thermophoresis, thermal radiation, viscous dissipation and Joule heating respectively (see ref. [15] of the manuscript). Thus we can take any value of these parameters in the range 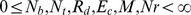. The larger the values of these parameters the greater will be the corresponding effect. In absence of magnetic field 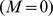, there is a decrease in 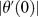 and increase in 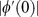 with an increase in the viscous dissipation effect. These variations increase with the higher values of Prandtl number (i.e.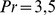), as can be observed from Table 2. This is primarily due to the fact that the Prandtl number controls the relative thickness of the momentum and thermal boundary layers. Therefore increasing values of 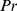 reduces conduction but enhances pure convection as well as variations in thermal characteristics due to viscous dissipation or joule heating. It is seen that in the presence of viscous dissipation and Joule heating effects, heat flux at the wall decreases with an increase in 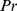. This outcome is in accordance with Makinde et al. [35]. In Table 3, the computed values of 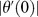 and 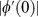 are given for various values of 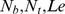 and 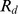 with the other parameters fixed. It is found that in absence of thermal radiation there is significant reduction in wall temperature gradient when 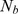 and 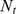 are increased and this variation is smaller in the presence of thermal radiation.

**Table 1:** Comparison of current results with Kuznetsov and Nield [13] with 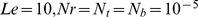 in the absence of 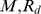 and 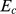.

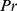		1	10	100	1000
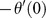	Kuznetsov and Nield [13]	0.401	0.463	0.481	0.484
	Present	0.401007	0.463285	0.481067	0.483602

**Table 2:** Variation of Nusselt number (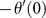) and Sherwood number (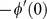) when 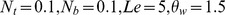 and 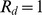.

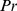	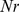	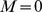				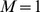			
		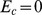		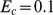		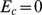		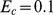	
		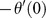	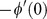	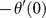	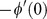	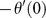	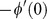	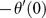	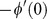
1	0	0.1925	0.8844	0.1812	0.8941	0.1542	0.7479	0.1422	0.7572
	0.5	0.1861	0.8370	0.1773	0.8449	0.1464	0.6875	0.1372	0.6958
	1	0.1784	0.7776	0.1719	0.7841	0.1361	0.6037	0.1296	0.6122
3	0	0.2201	0.9460	0.1720	0.9883	0.1618	0.7634	0.1176	0.7982
	0.5	0.2128	0.8992	0.1740	0.9355	0.1531	0.7013	0.1186	0.7332
	1	0.2042	0.8419	0.1742	0.8734	0.1418	0.6158	0.1167	0.6485
5	0	0.2305	0.9690	0.1363	1.0524	0.1635	0.7670	0.0786	0.8329
	0.5	0.2229	0.9226	0.1457	0.9954	0.1547	0.7044	0.0867	0.7660
	1	0.2140	0.8662	0.1530	0.9306	0.1431	0.6185	0.0920	0.6821

**Table 3:** Variation of Nusselt number (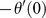) and Sherwood number (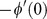) when 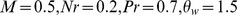 and 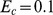.

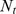	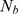	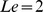				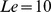			
		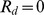		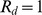		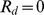		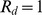	
		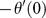	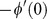	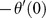	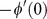	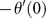	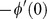	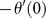	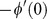
0.1	0.2	0.2738	0.4332	0.1516	0.5299	0.2686	0.9416	0.1528	1.0277
	0.5	0.2286	0.4770	0.1433	0.5379	0.2107	0.9653	0.1434	1.0292
	0.8	0.1879	0.4929	0.1352	0.5411	0.1634	0.9765	0.1344	1.0308
0.2	0.2	0.2624	0.3967	0.1494	0.5228	0.2570	0.9427	0.1508	1.0315
	0.5	0.2199	0.4712	0.1415	0.5366	0.2016	0.9751	0.1416	1.0324
	0.8	0.1807	0.4938	0.1336	0.5412	0.1562	0.9872	0.1328	1.0337
0.3	0.2	0.2517	0.3665	0.1473	0.5163	0.2461	0.9489	0.1489	1.0353
	0.5	0.2117	0.4674	0.1398	0.5356	0.1930	0.9861	0.1398	1.0357
	0.8	0.1738	0.4958	0.1320	0.5414	0.1494	0.9983	0.1312	1.0367

**Figure 2:**
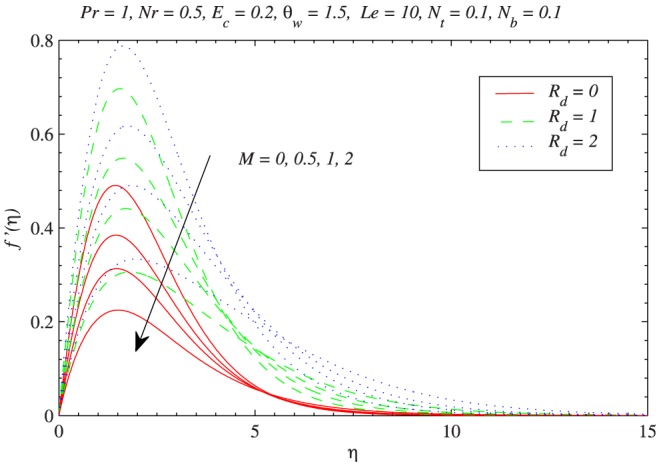
Influence of magnetic field on the vertical component of velocity 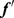 with the variation in radiation parameter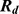.

**Figure 3:**
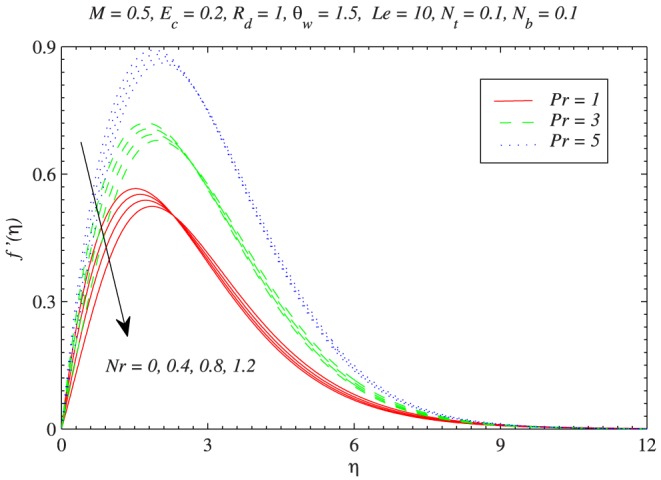
Influence of buoyancy force on the vertical component of velocity 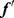 with the variation in Prandtl number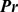.

**Figure 4:**
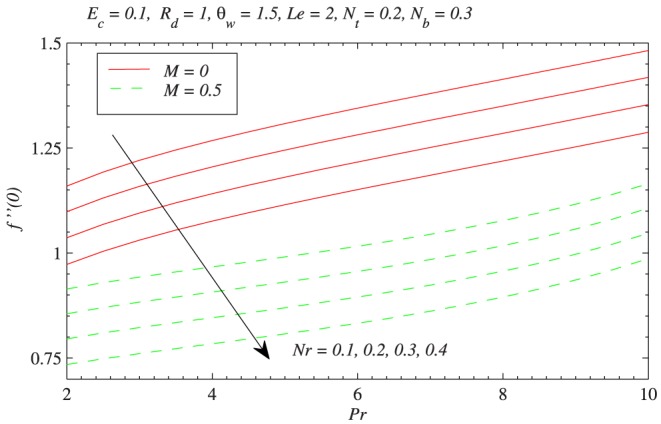
Influence of buoyancy force on the wall shear stress 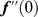 with the variation in magnetic field strength.

**Figure 5:**
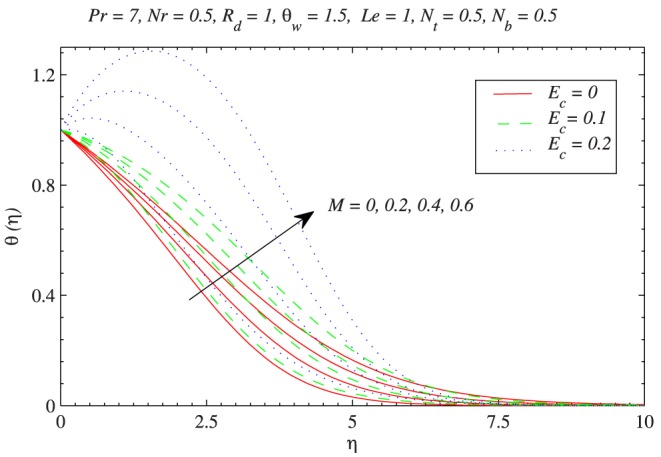
Influence of magnetic field on the temperature distribution with varying viscous dissipation effect.

**Figure 6:**
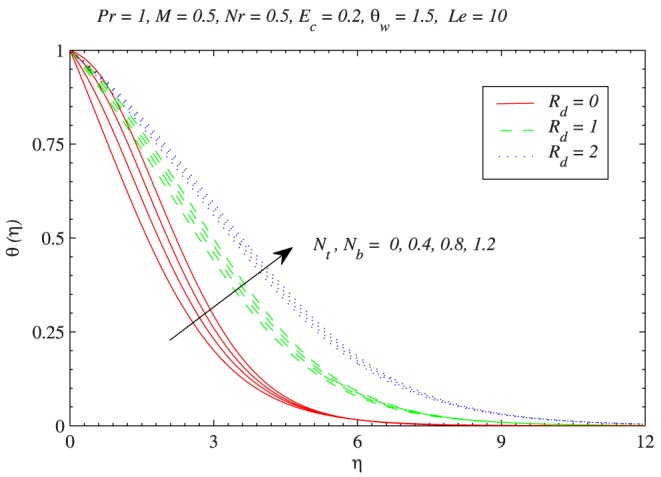
Combined influence of Brownian motion and thermophoretic diffusion on temperature distribution with the variation in thermal radiation effect.

**Figure 7:**
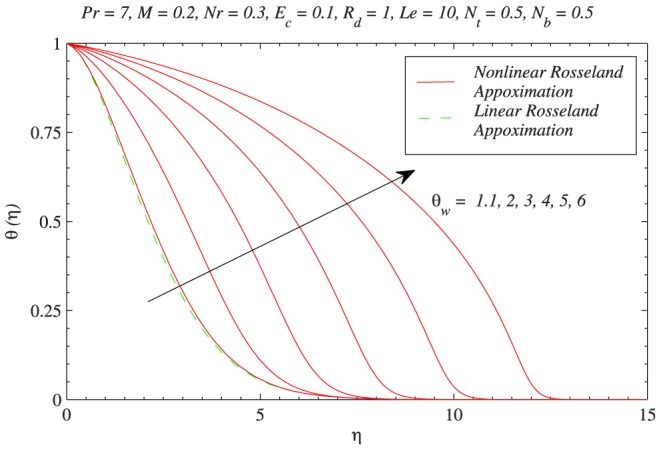
Influence of temperature ratio parameter 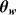 on temperature profiles and a comparison between linear and nonlinear radiation heat transfer.

**Figure 8:**
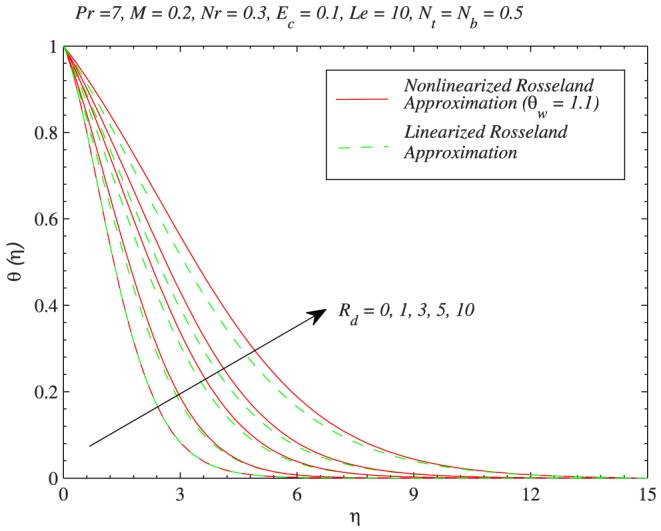
Effects of thermal radiation on the temperature distribution through both linear and non-linear radiative heat fluxes when 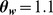.

**Figure 9:**
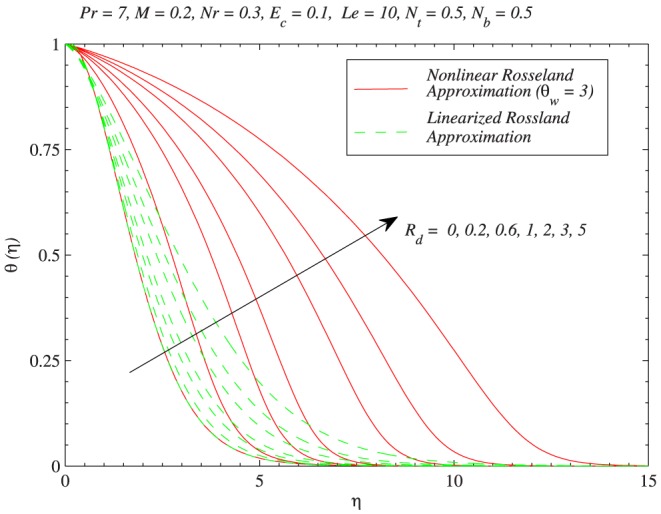
Effects of thermal radiation on the temperature distribution through both linear and non-linear radiative heat fluxes when 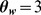.

Fig. 2 describes the influences of radiation and magnetic field on the dimensionless velocity profiles. The vertical (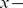 component) of velocity increases to a maximum value and asymptotically reaches to zero value near the edge of boundary layer. Fig. 2 also indicates that hydrodynamic boundary layer is thicker at 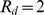 than at 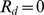. On the other hand the boundary layer becomes thinner when magnetic field strength is increased. The decrease in momentum transport is due to the presence of Lorentz force induced by the applied magnetic field perpendicular to the flow. Fig. 3 shows the variations in velocity profiles with an augmentation in 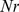 for three different values of 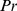. In accordance with Kuznetsov and Nield [13] and Aziz and Khan [31], as Prandtl number is increased from 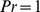 to 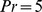 the hydrodynamic boundary layer becomes thicker. Further a decrease in the vertical component of velocity with an increment in 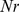 is accounted and this decrease is of similar magnitude for 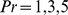. It is worth mentioning here that 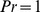 corresponds to the electrolyte solution such as salt water. The Prandtl number between 4 and 5 represents R-12 refrigerant. For water-based nanofluid the Prandtl number is between 6 and 7. Physically when 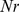 is increased, the fluid is under the influence of stronger buoyancy force which reduces the vertical component of velocity. Fig. 4 plots the wall velocity gradient versus 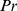 with the variation in 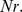 The results are presented for three different values of 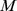. It is clear that wall shear stress can be decreased by either increasing the strength of magnetic field or by increasing the buoyancy-ratio parameter 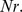 The behavior of Joule heating on the thermal boundary layer can be visualized from Fig. 5. 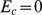 corresponds to the case when there is no viscous dissipation effect. For a weaker viscous dissipation effect i.e. when 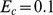, there is a minor growth in the thermal boundary layer thickness with an increase in 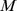 and this variation increases as viscous dissipation effect is strengthened. It is seen that for stronger viscous dissipation effect, the resistance in the fluid motion caused by the Lorentz force and by friction enhances the temperature. As viscous dissipation effect gets strengthened (i.e. 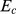 changes from 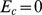 to 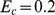) the fluid’s temperature near the vicinity of the plate becomes greater than the plate’s temperature which results in reverse heat flow close to the plate as can be seen from Fig. 5. We therefore expect the reduced Nusselt number 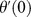 to be negative in this situation. The influence of Brownian motion and thermophoresis parameters on the temperature profiles is presented in Fig. 6. Increase in the Brownian motion leads to significant movement of nanoparticles within the base fluid which eventually increases the fluid’s kinetic energy and thus temperature rises. The thermal boundary layer also becomes thicker as thermophoretic effect is increased. Interestingly the variations in temperature function with 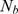 and 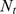 becomes less when radiation parameter 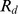 is increased from 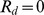 to 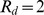. In Fig. 7, the green line is obtained from the solution of Eq. (11) (which is for linear radiation problem) and red lines are computed through Eq. (12) with the variation in 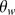. Increasing values of 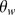 indicates larger wall temperature compared to ambient. As a result temperature 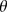 is an increasing function of 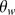. Moreover the profiles become S-shaped (earlier pointed out by Pantokratoras and Fang [50]) indicating the occurrence of adiabatic case for sufficiently larger value of 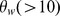. It is worth pointing here that when 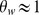, temperature profiles are close to corresponding profiles for the case of linear radiative heat flux. Figs. 8 and 9 compare the results of linear and non-linear radiation for different values of 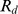 when 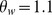 and 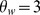 respectively. It is seen that linear and non-linear results match each other better at 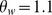 when compared with 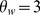. The profiles show a significant deviation as the radiation parameter is gradually increased, a fact that can be understood through a comparison of Eqs. (11) and (12). Thus it can be concluded that linear and non-linear radiation results match up smoothly when 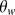 is close to one (say 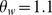) and 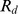 is sufficiently small (say 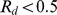).

**Figure 10:**
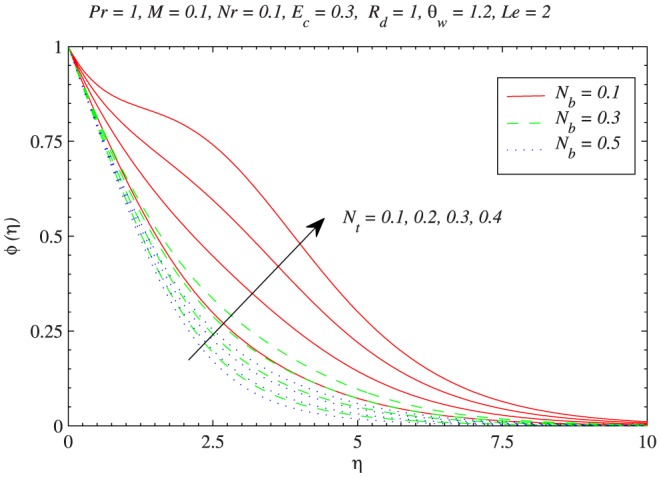
Influence of thermophoretic diffusion on nanoparticle concentration 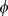 with the variation in Brownain motion.

**Figure 11:**
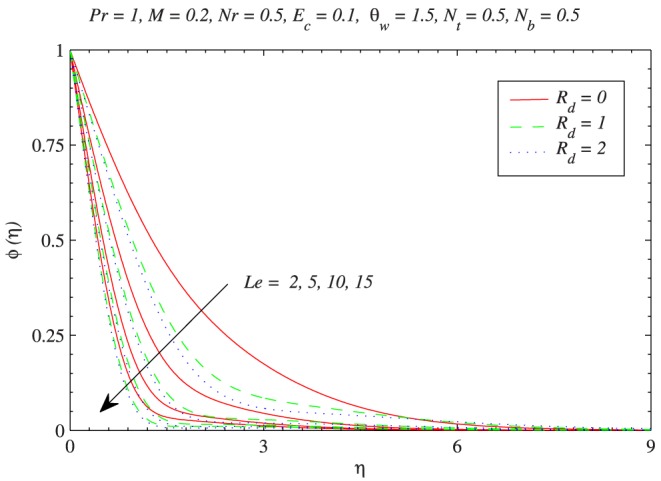
Influence of Lewis number 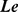 on the nanoparticle concentration 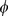 with the variation in thermal radiation effect.

Fig. 10 depicts the thermophoretic effect on the nanoparticle volume fraction 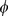. For hot surfaces, thermophoresis tends to blow the nanoparticle volume fraction boundary layer away from the surface since a hot surface repels the sub-micron sized particles from it, thereby forming a relatively particle-free layer near the surface. As a consequence, the nanoparticle distribution does not exist closer to the plate. It can also be seen that there is a significant increase in 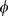 with an increase in 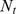 for a weaker Brownian motion 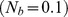. The change in nanoparticle volume fraction profiles with an increase in 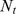 becomes smaller as the Brownian motion strengthens i.e. when 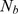 changes from 0.1 to 0.5. The behavior of Lewis number 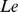 on 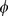 can be interpreted from Fig. 11. Increasing values of 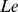 corresponds to a decrease in mass diffusivity or less Brownian diffusion and eventually less penetration depth for concentration boundary layer. It is noticeable that values of 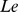 around 0.3–1.62 represent various gases which include hydrogen, methane, ethylene and propane. Moreover the concentration 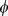 is a decreasing function of 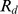.

**Figure 12:**
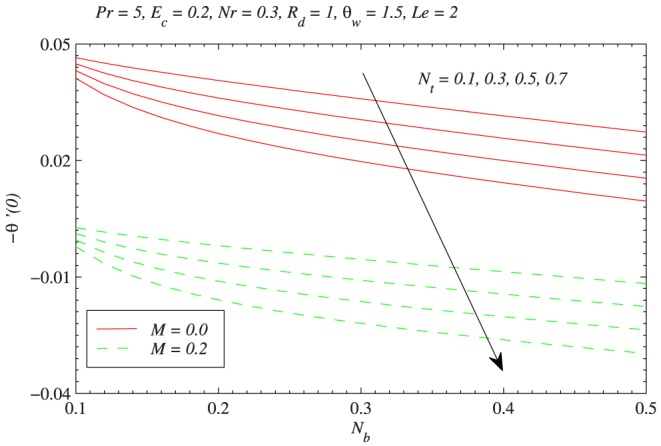
Influence of Brownian motion and thermophoretic diffusion on wall heat transfer rate 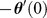.

**Figure 13:**
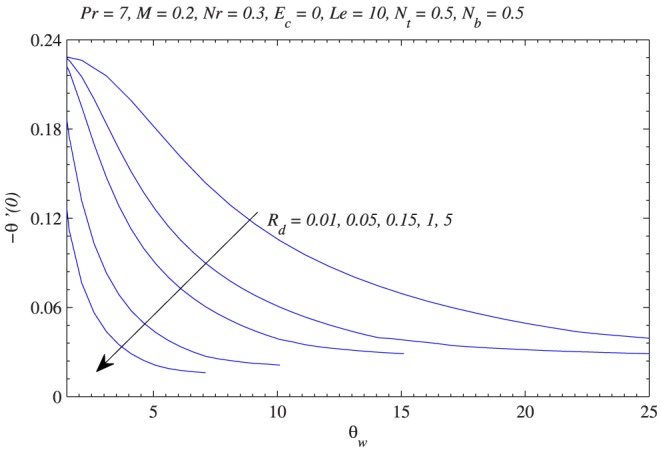
Influence of temperature ratio parameter on wall heat transfer rate 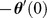 with the variation in radiation parameter.

**Figure 14:**
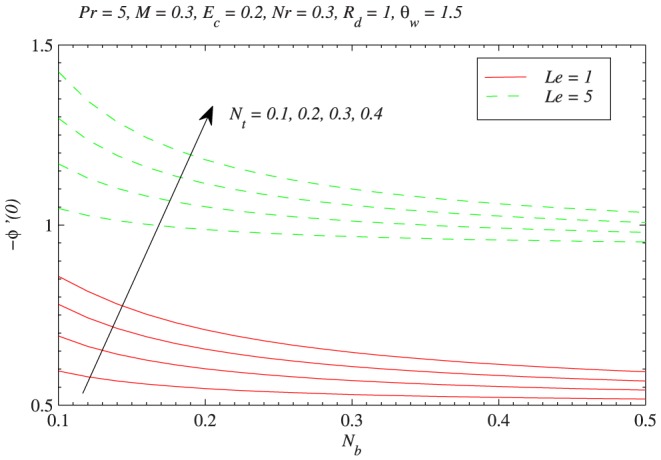
Influences of Brownian motion and thermophoresis on nanoparticle wall mass flux with the variation in Lewis number.

Fig. 12 shows the variation in reduced Nusselt number with 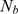 for different values of 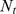. This Fig. is complimenting the data shown in Table 2. It is seen that 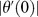 is a decreasing function of 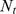 and this decrease is of similar magnitude for any value of 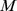 and 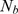. Physically a stronger thermophoretic force drives the nanoparticles from the plate to the fluid invoking a particle-free layer near the plate. Fig. 13 plots the wall temperature gradient versus 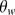 for different values of radiation parameter 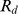. When 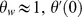 tends to a constant value for sufficiently smaller values of 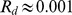 which is in accordance with Cortell [52]. Fig. 14 indicates that variation in 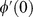 with Brownian motion and thermophoresis effects is similar for 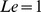 and 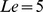. It should be noted here that behaviors of parameters on the velocity, temperature, local nanoparticle volume fraction and heat transfer rate discussed above are in accordance with the previous studies [12], [13], [20], [21] and even with Kuznetsov and Nield [33] about the revised boundary condition for nanoparticle volume fraction.

## Conclusions

The present work investigates the influence of nonlinear thermal radiation on the natural convective boundary layer flow of an electrically conducting nanofluid past a vertical plate. Different from the linear radiation problem, the present problem is governed by an additional temperature ratio parameter. Mathematical model incorporates the effects of Joule heating and viscous dissipation in the energy equation. The solutions are computed numerically by shooting method with fourth-fifth-order Runge-Kutta integration technique. The major conclusions are listed below:

- Temperature increases and profiles become S-shaped with an increase in temperature ratio parameter and decrease in thermal radiation parameter revealing the existence of the point of inflection for temperature distribution.
- Unlike previously reported results on thermal radiation effect, current results are valid for both small 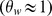 as well as large temperature differences 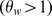 between plate and environment.
- Temperature 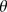 increases and thermal boundary layer thickens when 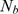 and 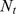 are increased. The variation in temperature function is of smaller magnitude for a stronger thermal radiation effect.
- Increase in the strengths of Brownian motion and thermophoresis effects results in the decrease of heat transfer from the plate.
- Temperature 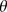 increases and heat transfer from the plate decreases by increasing Joule heating and viscous dissipation effects.
- The points c, d and e still hold in case of linear radiation and differences in the behaviors of parameters (except the radiation parameter) are only quantitative.

With the inclusion of non-linear radiation term, which allows both small and large temperature differences in the flow, the current work may lead to better understanding of the absorption of incident solar radiation and its transition into the nanoparticle working fluid. Further natural convection in nanofluids has vital importance in next generation solar film collectors, nuclear reactor application, heat exchangers etc.
